# Stenting in Obstructive Jaundice: ERCP VS PTC—no Final Answer

**DOI:** 10.1155/1989/86357

**Published:** 1989

**Authors:** Phillipus C. Bornman

**Affiliations:** Department of Surgery and Surgical Gatroentrology Groot Schuur Hospital and University of Cape Town Cape Town South Africa


misconceptions are true today. The results of the study being reviewed emphasises the importance of such patients being referred to major centers.

In my opinion the morbidity rates can be improved significantly even though most of the complications were minor. Operative mortality should be below 5% in expert hands. When one notes that 30% of the patients with solitary tumours and as many as 18% of the patients with multiple tumours survived 5 years this provides convincing evidence favouring liver resection. Note that the resection of colorectal metastases in the liver is probably more justified than surgery for many other types of malignant lesions including primary surgery for gastric or pancreatic carcinoma. I hope that an awareness of these results will change the referral pattern in many areas of the world.

S. Bengmark
Dept of Surgery
Lund University
221 85, Lund, Sweden

### REFERENCE

1. Ekberg, H., Tranberg, K-G., Andersson, R., Lundstedt, C., Hagerstrand, I., Ranstamn, J. and Bengmark, S. (1986) Determinants of survival in liver resection of colorectal secondaries. *British Journal of Surgery*, **73**, 727–731.

### STENTING IN OBSTRUCTIVE JAUNDICE: ERCP VS PTC — NO FINAL ANSWER

*A.G. Speer, P.B. Cotton, R.C.G. Russel, R.R. Mason, A.R.W. Hatfield, J.W.C. Leung, K.D. Macrae, J. Houghton, C.A. Lennon. (1987) Randomised trial of endoscopic versus percutaneous stent insertion in malignant obstructive jaundice. Lancet ii, 57–62.*

### ABSTRACT

**Patients with biliary obstruction due to malignant disease, and judged unfit for open operation, were randomised to have a biliary stent inserted either endoscopically via the papilla of Vater or percutaneously. Analysis after 75 patients had been entered showed that the endoscopic method had a significantly higher success rate for relief of jaundice (81% versus 61%, p = 0.017) and a significantly lower 30-day mortality (15% versus 33%, p = 0.016). The higher mortality after percutaneous stents was due to complications associated with liver puncture (haemorrhage and bile leaks). When stenting is indicated in elderly and frail patients the endoscopic method should be tried first.**

### ESSAYIST'S SUMMARY

75 patients with malignant biliary obstruction deemed unfit or technically unsuitable for a biliary bypass operation were randomised to have a biliary stent inserted either endoscopically (EP) or via the percutaneous transhepatic route (PTE).

Patients were referred from many hospitals in and around London but stents were inserted at only two centres (The London and Middlesex Hospitals by gastroenterologists and radiologists with considerable experience in either techniques.

The two treatment groups, EP 39 patients and PTE 36 patients were comparable with regard to age, ASA grading and other important risk factors in jaundiced patients. More patients with hilar lesions however were randomised to EP. Histological confirmation was obtained in 65% of the patients.

Endoscopic stenting proved to be superior to PTE in several respects; (1) initial successful placement (EP 89%; PTE 76%) (2) effective drainage, (EP 30/37 (81%), PTE 20/33 (61%). $p = 0.017$). (3) early complications (EP 7 (19%) of 37 patients and PTE 22 (67%) out of 33 patients) and (4) 30 day mortality (EP 15% versus PTE 33%). Nine of the 13 patients in the PTE group where drainage failed developed complications and there were 9 deaths amongst these patients. The 7 failures in the EP group led to 1 complication and 2 deaths.

The overall median survival in the two groups was short (EP 119 days; PTE 88 days. NS), particularly amongst patients with hilar lesions (EP 65 days, PTE 24 days). The survival was appreciably better in those patients with successfully placed stents (EP· 159 days and PTE 113 days). There was no difference in the stent patency rate in the two groups.

### PAPER DISCUSSION

**Keywords:** Endoprosthesis; jaundice, obstructive; biliary stent.

The development of non-operative stenting techniques has initiated a new era in the treatment of advanced malignant biliary obstruction. Patients who would normally have been turned down for bypass surgery because of extensive disease, or who are unfit for an operation, can now be offered biliary decompression by either the percutaneous transhepatic or the endoscopic route. Endoscopic stenting, the more recent of the two techniques, was initially limited to the standard duodenoscope which allowed placement of only small size stents (5–7F), and was associated with a high incidence of blockage and cholangitis[1]. The situation has now changed with the availability of the large channel (3.8 mm and 4.2 mm) duodenoscopes. In uncontrolled studies endoscopic stenting in expert hands has yielded similar results to PTE[2,3], and has beome a serious contender in the management of these patients.

The study by Speer and colleagues, the first controlled study comparing these two stenting techniques, concluded in favour of EP and thus seems to substantiate claims that this is the safer procedure. The results of endoscopic stenting in this study are most enviable and reflect the expertise of this group who are acknowledged world authorities with this particular technique. On the other hand, their complication rate for PTE is the highest published to date, and was mostly related to bile leakage. Bile leakage was responsible for 10 of the 19 complications (3 of 8 failures and 7 of 25 successful insertions) and six (60%) of these patients died.

Bile leakage after failed stenting is understandable but it is not clear why 7 of the 25 patients who had "successful placement of stents" developed this complication. In our controlled study[4] the incidence of bile leakage was significantly lower (1 of 25.$p<0.05$ Fisher's 2 tail) and is probably attributable to routine embolisation of the liver puncture tract with an "Ivalon" pledget (polyvinyl alcohol : Cook inc) – a technique not used in the present study. While the high incidence of complications may in part be explained by careful documentation, it is conceivable that the endoscopic experts had an advantage over their PTE counterparts.

This paper highlights the problem of interpretation of studies where technical expertise is a major determining factor in the outcome of results, even in a controlled trial setting. The results produced by referral centres may also be skewed by the referral pattern. We are not told what investigations patients underwent before they were referred for treatment but, it is possible, that a degree of selection took place in terms of suitability for stenting. By the same token, the timing of randomisation may also have had an influence on the selection of patients for the trial. With these provisos, the data presented by Speer and colleagues support their conclusion regarding the advantages of endoscopic stenting over PTE. Endoscopic stenting is safer and has fewer complications, even when the procedure fails, and the stent is easier to replace when blockage occurs. However, the availability of expertise and the complimentary use of both techniques in a centre, should ensure the best chance of successful outcome.[5].

The selection of patients with advanced disease for palliative stenting remains difficult. Many survive for only a short period and in those where the procedure fails, complications prolong hospital stay and lead to early death. Further studies are clearly needed to identify the risk factors for complications and those patients who would benefit most from stenting. No clear guidelines are currently available but it would seem ill-advised to stent patients with associated ascites (in the case of PTE), those with extensive lesions involving the intrahepatic ducts and when the necessary expertise with a particular technique is not available. Many patients without associated pruritus may well be better off with appropriate supportive measures without stenting. In the light of current knowledge such a decision should not evoke ethical problems.

Phillipus C Bornman
Department of Surgery and Surgical Gastronterology
Groote Schuur Hostpital and University of Cape Town
Cape Town, South Africa

### REFERENCES

1. Siegel, J.H. (1984) Improved biliary decompression with large caliber endoscopic prostheses. Gastrointest Endosc; **30**: 21–23.
2. Huibregtse, K., Tytgat, G.N. (1982) Palliative treatment of obstructive jaundice by transpapillary introduction of large bore bile duct endoprosthesis. *Gut;* **23**: 371–375.
3. Huibregtse, K., Katon, R.M., Coene, P.P., Tytgat, G.N.J. (1986) Endoscopic palliative treatment in pancreatic cancer. *Gastrointest Endosc;* **32**: 334–338.

4. Bornman, P.C., Harries-Jones EP, Tobias, R., Van Stiegmann, G., Terblanche, J. (1986) Prospective controlled trial of transhepatic biliary endoprosthesis versus bypass surgery for incurable carcinoma of head of pancreas. *Lancet;* i: 69–71.
5. Robertson D.A.F., Ayres R., Hacking, C.N., Shepherd, H., Birch, S., Wright, R. (1987) Experience with a combined percutaneous and endoscopic approach to stent insertion in malignant obstructive jaundice. *Lancet;* ii: 1449–1452.

### SELECTED HPB INTERNATIONAL

### *INSTRUCTIONS TO AUTHORS*

This section aims to provide the readers of *HPB Surgery* with a selected abstracting service with a difference. The Editor, the Editorial board of *HPB Surgery* and local colleagues will identify and select important and seminal papers in the field of hepatic, pancreatic and biliary surgery published in other major journals. Readers are also encouraged to contact the Editor of this section with suggestions on papers to be included. An authority in the field will be asked to comment in a signed short essay or editorial on the selected papers. Both the original abstract (or summary of the abstract) and the expert essay will be published.

The essay should be approximately two printed page (four to five double typewritten pages) and contain a few selected references. It is hoped that the experts will usually applaude but sometimes criticise the paper and will add relevant comment. The format will be left to the individual essayist.

The original authors will not be asked to comment on the essay, but are invited to submit comments, if they wish, for a special section entitled "Selected HPB International Correspondence". Here they will be afforded early publication. The Editor believes that in most instances there will be no need for the authors to reply.

The essay must conform to the style of *HPB Surgery* (see instructions to authors).

Readers comments on the aims and format of the Selected HPB International will be welcomed by the section editor.

